# Student and Teacher Culture and Composition and the Development of Gender Role Attitudes among Young Adolescents

**DOI:** 10.1007/s10964-023-01897-1

**Published:** 2023-11-13

**Authors:** Ricarda Ullrich, Mieke Van Houtte, Michael Becker

**Affiliations:** 1 Department of Educational Research and Educational Psychology, Leibniz Institute for Science and Mathematics Education (IPN), Olshausenstraße 62, 24118 Kiel, Germany; 2 Department of Educational Governance, DIPF | Leibniz Institute for Research and Information in Education, Rostocker Straße 6, 30323 Frankfurt am Main, Germany; 3 Department of Sociology, Ghent University, Sint-Pietersnieuwstraat 41 T1, 9000 Ghent, Belgium; 4 Center for Research on Education and School Development (IFS), Technical University Dortmund, Vogelpothsweg 78, 44227 Dortmund, Germany

**Keywords:** Gender role attitudes, Teacher culture, Student composition, Development

## Abstract

Research has shown that gender role attitudes develop during adolescence; however, the relevant predictors remain a matter of debate. In adolescence, the school environment gains in importance. Thus, the present study investigates how students’ and especially teachers’ culture and composition predict the development of gender role attitudes in young adolescents. The study addresses this question using a sample of 7360 Flemish students (44.8% girls), who were surveyed three times after entering secondary education between 2012 (*M*age = 13.14, *SD* = 0.56) and 2014. Latent change models reveal that boys’ initial gender role attitudes are associated with the students’ gender role culture; however, boys with more traditional gender role attitudes do not develop in an even more traditional direction at the beginning of secondary education. In contexts with a more privileged student SES composition, boys develop less traditional attitudes, while a traditional gender role culture among teachers supports the development of more traditional gender role attitudes among boys. Girls with more traditional gender role attitudes find themselves within student contexts with a more traditional culture. However, the development does not vary with the students’ gender role culture. Overall, boys seem more susceptible to students’ cultural and compositional characteristics.

## Introduction

Attitudes towards gender roles and how they are lived out are subject to change, both at the societal level and at the level of the individual life course (e.g., Dotti Sani & Quaranta, 2017). Adolescents’ gender role attitudes are especially amenable to development, as gender-related constructs are salient during adolescence; this is because teenagers are experiencing biological, cognitive and social changes that can affect their attitudes (e.g., Ruble & Martin, 2006). Despite the relevance of these processes, few studies have examined the development of gender role attitudes during adolescence and relevant predictors of this development, while the findings are quite contradictory. Research has shown that parents’ gender role attitudes and socioeconomic status (SES) are relevant predictors of the extent of traditional gender role attitudes (Halimi et al., 2016). However, they are less suitable to predict the development of these attitudes (e.g., Halimi et al., 2021; Ullrich et al., 2022). It is generally known that the importance of the family context decreases during adolescence, and the importance of peer groups and the school context increases (Halimi et al., 2021). Nevertheless, there is very little, if any, research that deals with the school contexts’ impact on the development of gender role attitudes, although the school context is perceived as a critical socialization context during adolescence (Bronfenbrenner, 1977). Teachers are often overlooked, even though they are important socialization agents in school contexts (Van Houtte, 2011). Therefore, this study aims to test for the first time how students’ gender role culture, sex composition, and SES composition, along with teachers’ gender role culture and sex composition, can predict the development of gender role attitudes in early adolescence.

### The Development of Gender Role Attitudes During Adolescence

As a social construct of gender, gender roles consist of normative expectations about the distribution of power and work between the sexes. Gender roles refer to the relationship between men and women in terms of romantic partnerships, family divisions of labor, and professional careers, which is defined by a cultural and historical context. Traditionally, gender roles were assigned on the basis of binary sex, with the male role being associated with the task of family breadwinner and the female role being linked to social and domestic activities (e.g., Eagly & Wood, 2012). However, this traditional division is eroding, and an egalitarian model is emerging. In an egalitarian model, domestic and professional activities are distributed without reference to gender (e.g., Scarborough et al., 2019). Children and adolescents are exposed to these gender roles and develop their own attitudes towards gender-related role distributions through observation. These attitudes change over the lifespan and shift considerably during adolescence, as adolescents experience cognitive, social, and biological changes that may affect their attitudinal concepts (e.g., Blakemore et al., 2013). It is argued that the process of cognitive maturation and exploring their sexuality may cause young adolescents to question moral constructs and re-evaluate their assumptions about gender roles (Eccles, 1987). This leads to the avoidance of cognitive dissonance in adolescents (e.g., women can have professional careers and be good mothers), as adolescents can distinguish between prescriptive and descriptive gender norms and create cognitive representations of newer, more complex social arrangements (e.g., Eccles, 1987). Simultaneously, adolescents are discovering their sexual identity, undergoing hormonal and physical changes, and experiencing their first romantic relationships. Accordingly, the *gender intensification hypothesis* assumes that gender role behavior intensifies during adolescence, as young people learn to incorporate their later adult roles, including their gender roles, through early experiences with romantic relationships. This implies that traditional attitudes towards gender roles would intensify during early adolescence due to increased socialization pressures (Hill & Lynch, 1983).

Due to the different implications gender roles have for men and women, gender role attitudes of adolescent boys and girls may differ as well. Girls in particular might be aware of the implications and limitations the traditional female role entails, which is why egalitarian gender roles might be especially relevant for them (Thijs et al., 2019). Previous research found a decline in traditional gender role attitudes among female Mexican-American adolescents aged between 13 and 20 (Updegraff et al., 2014). This pattern was also found for Flemish adolescent girls between 7^th^ and 8^th^ grade, while traditional gender role attitudes for boys increased (Halimi et al., 2021). However, a few studies found no different developmental trajectories between male and female adolescents: A German study on the development of egalitarian gender role attitudes showed an increase in egalitarian gender role attitudes for female and male adolescents over the whole course of adolescence (Ullrich et al., 2022). In line with this, research concerning the development of traditional gender role attitudes has shown declines for female and male African-American adolescents aged between 9 and 18 (Lam et al., 2017) and Mexican-American adolescents aged between 11 and 17 (Schroeder et al., 2019).

Trying to explain these developmental trajectories, studies have looked at different predictors at the individual level. For example, research found that parents’ gender role attitudes (Halimi et al., 2021; Schroeder et al., 2019) and familial SES (Halimi et al., 2021; Schroeder et al., 2019; Ullrich et al., 2022) are associated with the extent of gender role attitudes but they are less able to predict their development. As adolescents’ distance themselves from the family environment, peer groups gain in importance. In line with that, previous research showed for both boys and girls that individually perceived peer pressure for gender conformity is associated with more traditional initial attitudes in young adolescents but affects the development of traditional attitudes negatively (Halimi et al., 2021). Additionally, early adolescents’ perceived peer pressure was positively associated with the own-gender typicality (perceived similarity to own gender group, Cook et al., 2019).

Research shows that gender role attitudes are salient during adolescence. However, developmental trajectories remain a matter of debate. Research attempting to explain different developmental patterns referred to individual-level predictors, while contextual factors remained unaddressed. Gender development is perceived as an interaction between the individual and their socialization environment (Blakemore et al., 2013; Eccles, 1987; Hill & Lynch, 1983). Along with the family environment, the school context with the student body and teachers are considered the most important socialization context during adolescence (Bronfenbrenner, 1977). However, the school context has not yet been studied with regard towards the development of gender role attitudes.

### Student Culture and Composition and the Development of Gender Role Attitudes

Adolescents’ central contextual environments are the family, peer groups, and the school. In the transition to adolescence, the school context is considered a critical social environment (Bronfenbrenner, 1977). Peer acceptance and popularity gain in importance during adolescence and teenagers seek acceptance, belongingness, and social integration, which can be gendered. These include gender norms and avoidance of rejection — it may be easier for adolescents to align with perceived norms about gendered roles (Cook et al., 2019; Huyge et al., 2015). A gendered student culture can be understood as shared assumptions, meanings, or beliefs within a school (e.g., Huyge et al., 2015). Research has shown that the variance in gender role attitudes is greater between schools than within schools, implying that there is a gender role-based school culture that varies with school composition characteristics (Van Houtte, 2021; Vantieghem, 2015). The school environment channels conceptions of masculinity (Legewie & DiPrete, 2012) or machismo (Huyge et al., 2015) which can be associated with the development of attitudes and behavior. Previous research showed that a more traditional gender role culture in a school is associated with more traditional gender role attitudes among students at the individual level (Halimi et al., 2020). However, the impact of gender role culture on the development of gender role attitudes has not yet been studied. As developmental trajectories can be different for boys and girls, predictors of this development might also affect girls and boys differently. Boys are more susceptible to the felt pressure for gender conformity (e.g., Vantieghem & Van Houtte, 2015); it is possible that boys are particularly sensitive to the schools’ gender role culture. However, even though girls seem to be not as susceptible as boys, a more open gender role environment should be especially relevant for girls because egalitarian structures are essential for offering girls the same opportunities as boys.

Since girls and women exhibit more egalitarian gender role attitudes than boys and men, the gender role culture in schools tends to be more open and egalitarian when there is a higher proportion of female students and teachers (e.g., Vantieghem, 2015). Research has shown that having a higher proportion of female friends is associated with less traditional gender role attitudes in girls (Halimi et al., 2021) and that girls in single-sex schools express more egalitarian gender role attitudes than girls in mixed-sex schools (Erarslan & Rankin, 2013). Since girls generally hold more egalitarian gender role attitudes, it could be assumed that with a higher proportion of girls in a school, girls develop in a less traditional direction because they are exposed to more open and egalitarian attitudes. At the same time, the pressure to behave in a gender-conforming manner felt by boys may increase with the proportion of girls in a school and when boys perceive themselves as a minority. This correlation is particularly evident when boys show more traditional attitudes (Van Houtte, 2021). On the other hand, it could be assumed that especially in male-dominated contexts traditional gender roles are supported (Jackson, 2002), which leads to more traditional gender role attitudes, especially for boys. Thus, the sex composition of the student body could have differential effects for girls and boys.

In addition to its sex composition, the SES composition of the student body is of relevance. Concerning parental SES, research has shown that SES is closely linked to students’ aspirations, their school and career trajectories (Stocké et al., 2011), and their expression of more egalitarian gender role attitudes (e.g., Ullrich et al., 2022). This is especially relevant for girls, as higher aspirations can only be realized through egalitarian labor market participation (e.g., Mays, 2012). Research has shown that a more privileged parental SES is associated with less traditional gender roles for both genders but is less predictive of the development of gender role attitudes (Halimi et al., 2021; Ullrich et al., 2022). Along with the school context becoming more relevant, the SES composition of a student body in a school could be especially significant for adolescents’ gender role attitudes. Research has shown that next to educational outcomes (e.g., van Ewijk & Sleegers, 2010), developmental outcomes such as sense of futility (Agirdag et al., 2012) or motivation (Hornstra et al., 2015) are associated with SES composition of the school. Previous research suggests that low SES school composition is particularly detrimental to boys’ educational participation, as it is associated with traditional masculinity and gender roles (e.g., Hadjar & Lupatsch, 2010). Assumingly, the schools’ gender socialization processes and therefore the development of gender role attitudes might vary with the SES composition. Related research has shown that girls in more privileged socioeconomic neighborhood schools have more egalitarian gender role attitudes (Erarslan & Rankin, 2013). However, the impact on boys and a developmental perspective have not yet been considered. Taken together, previous research indicates that schools’ gender role culture and sex composition is associated with adolescents’ individual attitudes towards gender roles, but a developmental perspective remains unexamined. Moreover, indirect evidence suggests that the SES composition of the school may be of relevance for gender role attitudes and their development during adolescence, which will be tested for the first time in the present study.

### Teacher Culture and Composition and the Development of Gender Role Attitudes

Teachers are often overlooked as important socialization agents in the school context. Teachers have internalized gender role attitudes, which can be transmitted through gendered expectations to the students (Wolter et al., 2015). Teachers’ gender role attitudes are related to how accepting they were of cross-gendered behavior and homosexuality among students (Cahill & Adams, 1997).

Different mechanisms have been discussed in how teachers’ gender-related attitudes are transmitted to children. This can occur through teachers’ gender-related expectations of students but also through modeling, as teachers can function as role models. The stronger teachers’ gender-related perceptions are, the more likely they are to treat students differently. Research showed that the more traditional teachers’ attitudes are, the less motivated boys feel to learn to read, because reading is stereotypically associated with girls (Wolter et al., 2015). Students use interests to regulate their gender identity; for instance, girls with higher gender typicality and higher pressure to conform to gender stereotypes show more interest in highbrow culture (Lagaert et al., 2017). Especially in gender-stereotyped subjects, students experience differential gender behavior from teachers, which can then become a self-fulfilling prophecy (Retelsdorf et al., 2015). Even when children interact with their teachers outside of direct instruction, gendered learning can occur, for example when emotional behavior is more likely to be tolerated in girls than in boys. Moreover, teachers themselves represent gender role models. If teachers are interested in gender-typical hobbies or if they teach gender-typical subjects, this can have an impact on students’ gender perceptions (Wolter et al., 2015). By being a role model and an expression of gender-related expectations, teachers can consciously or unconsciously reproduce the societal gender distribution, which they pass on to the next generation. However, students are not only exposed to one teacher — they are exposed to several teachers who act as role models. Research already showed that a certain culture of expectations evolves among teachers (Van Houtte, 2011) which can be understood as shared beliefs, norms and values (e.g., Skaalvik & Skaalvik, 2021). Indirect evidence for the importance of shared teachers’ beliefs is highlighted by research revealing that teachers’ shared beliefs regarding students’ teachability are associated with students’ deviant behavior (Demanet et al., 2019), the sense of academic futility (Agirdag et al., 2013), and perceived teacher support (Demanet & Van Houtte, 2012). In the sense of a gendered hidden curriculum (Basow, 2004), it can be assumed that teachers also share gender role attitudes to some degree and thus to the extent to which students’ gender-stereotypical behavior is accepted and tolerated in schools. Especially at secondary level, there are fewer one-to-one interactions. The use of teachers’ shared beliefs and their effect on students’ attitudes thus reflects the reality of secondary education (e.g., Van den Broeck et al., 2020). Hence, for the first time the present study examines how teachers’ gender role culture and sex composition are associated with the development of gender role attitudes of young adolescent boys and girls.

### Covariates

Studies have focused on different predictors at the individual level, thereby trying to explain different developmental trajectories of gender role attitudes during adolescence. Next to the school context, the most important socialization agents are parents and peer groups (Bronfenbrenner, 1977). Research on young Flemish adolescents shows that the parental SES is negatively associated with boys and girls traditional gender role attitudes in 7^th^ grade (Halimi et al., 2021). A German study confirms this pattern, where parental SES is positively associated with more egalitarian gender role attitudes of young adolescent boys and girls (Ullrich et al., 2022). In terms of peer groups, previous research shows that individually perceived peer pressure for gender conformity is associated with both the extent and the development of gender role attitudes (Halimi et al., 2021). The parental SES and the individually perceived peer pressure are therefore included as covariates in the analyses.

The Flemish school system is characterized by a rigid tracking system after six years of primary school when the students are about 12 years old. The tracking system is divided into four main tracks with a vocational, academic (classic languages or modern sciences), technical and art track. The tracking system is commonly organized between schools and only a few schools offer all four tracks or combinations of tracks. The decision for an educational track is made by the parents, supported by a recommendation from the teacher. Due to the tracking system the technical and vocational tracks are rather gender-specific leading to schools with predominantly boys or girls, while academic and art tracks are more gender mixed (for a detailed description of the context see Van Houtte & Vantieghem, 2020). Hence, the tracking system might be associated with adolescents’ gender role attitudes and will be included as covariates in the analyses.

## The Present Study

Since the school context and its teachers are crucial socialization agents, the present study addresses the extent to which the students’ gender role culture, sex composition, and SES composition, along with the teachers’ gender role culture and sex composition, can explain the development of gender role attitudes among male and female adolescents’ after moving into a new school context. First, the question will be answered how students’ gender role culture, sex composition, and SES composition are associated with young adolescents’ traditional gender role attitudes and their development. Accordingly, a more traditional gender role culture among students should be correlated with more initial traditional gender role attitudes in boys and girls and a development in a more traditional direction. A higher proportion of girls in a school should be associated with less traditional attitudes in girls and the development in a less traditional direction. As the theoretical assumptions for boys are contradictory, the present study examines the correlation and their development in an explorative way. The present study assumes that a more privileged SES composition among students is associated with less initial traditional gender role attitudes in boys and girls. Beyond that, and since the school context becomes particularly important during adolescence, students’ SES composition should be associated with boys and girls developing in a less traditional direction. Secondly, teachers are important socialization agents in terms of gender-related perceptions and behavior, which is why the question arises of how a traditional gender role culture among teachers and the sex composition of teachers are related to boys’ and girls’ gender role attitudes and their development during early adolescence. A traditional gender role culture among teachers could be associated with more initial traditional gender role attitudes among boys and girls and a development in a more traditional direction. As women express less traditional gender role attitudes, it can be assumed that a higher proportion of female teachers is correlated with less traditional gender role attitudes and a development in a less traditional direction.

## Methods

### Dataset

To answer the questions, the dataset *Teaching in the Bed of Procrustes* is used, which enabled an examination of the development of gender role attitudes across three waves, between Grades 7 and 8 (the first and second grade of secondary education). The data collection took place during the school years of 2012 to 2014 in Flemish secondary schools. Three random school samples were drawn based on three stratification characteristics and classified according to school denomination (public versus private), location (urban versus rural), and the geographical region within Flanders. If a school refused to participate in the study, a school with similar stratification criteria from the next random sample was contacted (Vantieghem, 2015). The data collection started in 2012, in the months of October and November at the beginning of secondary education (*M*age = 13.14, *SD* = 0.56). The second wave was conducted in April and May 2013 at the end of the first year of secondary education (*M*age = 13.64, *SD* = 0.67). The third and last wave was conducted one year later, in April and May 2014 (*M*age = 14.64, *SD* = 0.67). All students within the selected schools were asked to complete a paper-and-pencil questionnaire, with one researcher always being present to explain the procedure and to answer questions. As the study was deemed to be of minimal risk, children’s consent was approved by the school and the Belgian Commission for the Protection of Privacy. All teachers teaching in the first grade (that is the first and secondary year of secondary education in Flanders, 7^th^ and 8^th^ grade in the US) were invited to fill out the survey.

As the study is interested in the development of gender role attitudes, students were excluded when they had missing values on all gender role attitude items throughout the three measurement points (*N* = 155) and if sex was not recorded (*N* = 14). This led to a total sample size of 7360 students. Clustered in 59 schools, 6311 students participated in the first wave, 6158 in the second wave, and 6075 in the third wave (a more detailed attrition analysis is included in the sensitivity analyses). The study design guaranteed that students with different socioeconomic backgrounds and from several regions in Flanders were surveyed, which resulted in a gender distribution of 55.2% boys and 44.8% girls and 21.7% students with a non-Western European background (at least one parent was born in a non-Western European country). Thus, the sample can be regarded as representative in terms of gender proportions, SES, and students with a non-Western European background. The teachers were surveyed in the first wave and 1247 teachers responded. The dataset was particularly suitable examining the student development of gender role attitudes as the first data collection took place shortly after the transition to secondary education. Therefore, the change of school and peer context may have had an impact on the development of gender role attitudes.

### Measures

#### The student level

##### Students’ gender role attitudes

Students’ gender role attitudes were measured using a scale, which measured traditional gender role attitudes on a five-point Likert-scale ranging from 0 (completely disagree) to 4 (completely agree) (Vermeersch et al., 2010). The items’ contents are multidimensional and relate to gendered behavior in adult work and private life (e.g., “Children, not a career, are the main responsibility for a woman”), traditional masculinity (e.g., “A man without self-confidence is an idiot”), and gender perceptions of boys and girls (e.g., “There is definitely something wrong with a boy practicing ballet as a hobby”; for the original items in Dutch and their English translations, see Halimi et al., 2018). The gender role attitude scale was previously examined for reliability and validity using the *Teaching in the Bed of Procrustes* data (Halimi et al., 2018), resulting in 11 of 15 items being used, which were surveyed in all three waves. To achieve a sufficient model fit per wave (Table 2), one correlation on cross-gendered behavior in boys and girls (items 1: “I find it disturbing if a boy behaves like a girl” and item 5: “I find it disturbing if a girl behaves like a boy”) was allowed in all three waves. Table 1 displays the descriptive statistics for the latent gender role scales of all three measurement points, indicating that on average, boys expressed more traditional gender role attitudes than girls.

**Table 1:** Sample statistics for the complete sample and separately for boys and girls

	Overall sample		Boys		Girls	
	N	M (SD)	N	M (SD)	N	M (SD)
Gender role attitudes (t1)	6311	1.63 (0.61)	3394	1.81 (0.64)	2917	1.40 (0.50)
Gender role attitudes (t2)	6158	1.55 (0.75)	3298	1.85 (0.76)	2860	1.21 (0.58)
Gender role attitudes (t3)	6075	1.57 (0.82)	3293	1.95 (0.78)	2782	1.10 (0.62)
**School level**						
Students’ gender role culture	59	0.00 (0.23)	59	0.00 (0.23)	56	-0.01 (0.23)
Students’ sex composition	57	0.44 (0.21)	57	0.44 (0.21)	54	0.46 (0.19)
Students’ SES composition	57	4.70 (0.86)	57	4.70 (0.86)	54	4.73 (0.87)
Teachers’ gender role culture	58	0.00 (0.04)	58	0.00 (0.04)	55	0.00 (0.04)
Teachers’ sex composition	59	0.72 (0.14)	59	0.72 (0.14)	56	0.73 (0.14)
**Individual level**						
Parental SES	6147	5.05 (1.86)	3297	4.99 (1.86)	2850	5.13 (1.86)
Perceived peer pressure	6275	1.88 (0.30)	3372	2.02 (0.28)	2903	1.73 (0.24)

##### Sex

The sex of the students was binary-coded, with 0 for boys and 1 for girls. The sex of the first wave was used as a basis and missing values were filled with responses from the second and third wave.

#### The school level

##### Students’ gender role culture

To model the students’ gender role culture, the first measurement point was used as a reference for the school culture and the scale was tested for measurement invariance between the levels that reached scalar invariance (RMSEA = 0.04, CFI = 0.91, TLI = 0.91, SRMR within = 0.03, SRMR between = 0.18). From this factor, factor scores were extracted at the between level as an indicator of students’ gender role culture. The scale was previously tested as a measure of gender role culture and the results indicated that gender role attitudes varied more strongly between schools than within schools. This implies a shared gender role culture amongst students (Huyge et al., 2015; Van Houtte & Vantieghem, 2020). Factor scores are standardized scores with a mean of 0.00 (*SD* = 0.19, Table 1) and the students’ gender role culture ranged between −0.33 and 0.59, while higher scores indicate a more traditional school culture.

##### Students’ sex composition

The proportion of boys and girls in a school was measured by taking the proportion of girls in the first school year. The dataset contained schools with a percentage of 0% girls and schools with a percentage of 81% girls (*M* = 0.44, *SD* = 0.21).

##### Students’ SES composition

Likewise to the sex proportions, the mean SES composition of the schools was calculated (for the operationalization of parental SES, see the chapter on control variables). The SES proportions ranged from 2.57 to 6.59 with a mean of 4.70 (*SD* = 0.86).

##### Teachers’ gender role culture

Within the teacher survey at the first measurement point, teachers were asked to respond to the same gender role attitude scale as their students. As students are exposed to multiple teachers, at least five teachers per school were surveyed to reflect the general teacher culture. Teacher response rates varied from 4 to 56 teachers per school with a mean of 33.29 (*SD* = 17.14). Only the information from schools in which at least five teachers responded was considered appropriate for analysis (Van Maele & Van Houtte, 2009). To model teachers’ gender role culture, a latent multilevel model with measurement invariance between the levels was conducted. With an analog correlation between items 1 and 5 to the students’ gender role scale, the scale confirmed scalar invariance for the teachers’ gender role scale (RMSEA = 0.05, CFI = 0.93, TLI = 0.92, SRMR within = 0.04, SRMR between = 0.56). From the multilevel model with constraint factor scores and intercepts, factors scores on the between level were extracted as an indicator of teachers’ gender role culture. The teachers’ gender role culture ranged from −0.12 to 0.13 with a mean of 0.00 (*SD* = 0.04).

##### Teachers’ sex composition

The sex composition of teachers was also taken into account. This ranged from 32% female teachers in a school to 100% female teachers in a school (this applied to two schools with an all-female teaching staff). On average, female teachers made up 72% of schools’ teaching staff (*SD* = 0.14).

#### Control variables

##### Parental SES

To measure the parental SES, the highest employment status of both parents was used. To classify the occupations, the Erikson-Goldthorpe-Portocarero classification (EGP), was used which ranged from (1) unskilled manual labor to (8) high-grade professional manager (Erikson & Goldthorpe, 2002).

##### Peer pressure to gender conformity

To control for individually perceived peer pressure, the gender identity questionnaire was used (Egan & Perry, 2001). The scale consists of nine items that relate to the felt pressure to conform to gender norms. The students had to indicate whether they completely disagreed, disagreed, agreed, or completely agreed with statements like “Other [boys/girls] I know would be upset if I wanted to learn how to [sew or knit/fix cars or bicycles].” (for the original items in Dutch and their English translations, see Vantieghem & Van Houtte, 2015). The scale was latently modeled at the individual level and tested for measurement invariance between the genders. By allowing three correlations and releasing one restrained intercept, partial scalar measurement invariance between girls and boys was achieved (RMSEA = 0.06, CFI = 0.93, TLI = 0.93, SRMR = 0.06).

##### School tracks

Finally, to control for educational track, the school tracks were dummy coded with an art track (*N* = 57, 66.7% girls), a technical track (*N* = 1316, 34.5% girls), and a vocational track (*N* = 870, 33.4% girls) — the academic track (*N* = 3804, 52.5% girls) was used as the reference.

### Statistical analyses

Since the study is concerned with the development of gender role attitudes of adolescent boys and girls, it is important to ensure measurement invariance over time and between genders. This has hardly been tested in the developmental research on gender role attitudes but since gender roles change over time it is a crucial prerequisite for adopting a developmental perspective. Therefore, a latent factor structure of gender role attitudes for each measurement wave was implemented and tested for longitudinal and multigroup invariance by sex (Kim & Willson, 2014). This ensured that gender role attitudes could be assessed with the same scale and a uniform metric over a two-year period. It is essential to at least achieve scalar measurement invariance (constrained factor loadings and intercepts) to compare means on a common metric and to examine developmental questions (Meredith, 1993). To compare the model fit, the most common absolute measures were applied — RMSEA, CFI, TLI, and SRMR. These measures allowed us to evaluate the model fit independently of the sample size. Acceptance of the model fit was based on the following criteria: RMSEA < 0.08, CFI ≥ 0.90, TLI ≥ 0.90, SRMR < 0.08 (e.g., Rutkowski & Svetina, 2014). Longitudinal scalar measurement invariance was achieved by allowing autocorrelations (Table 2, RMSEA = 0.03, CFI = 0.95, TLI = 0.94, SRMR = 0.04). When testing the longitudinal measurement invariance for invariance between the sexes, one intercept over time had to be released for each gender. Over time, for girls, the intercept of item 8 was released (“Only slim girls are attractive to boys”), and for boys the intercept of item 6 was released (“There must be something wrong with girls who talk dirty”). These adjustments enabled us to achieve partial scalar measurement invariance over time and between boys and girls (Table 2, RMSEA = 0.03, CFI = 0.92, TLI = 0.92, SRMR = 0.04).

**Table 2:** Model fit indices of the gender role attitude scales and measurement invariance over time and between boys and girls

	N (boys/girls)	RMSEA	CFI	TLI	SRMR
CFA gender role attitudes (t1)^a^	6311	0.04	0.97	0.96	0.03
CFA gender role attitudes (t2)^a^	6158	0.05	0.97	0.96	0.03
CFA gender role attitudes (t3)^a^	6075	0.05	0.98	0.97	0.03
configural invariance over time^b^	7360	0.03	0.96	0.96	0.03
metric invariance over time	7360	0.03	0.96	0.96	0.04
scalar invariance over time	7360	0.03	0.95	0.94	0.04
scalar invariance over time and partial configural invariance between groups	4062/3298	0.03	0.92	0.92	0.04
scalar invariance over time and metric invariance between groups	4062/3298	0.03	0.92	0.92	0.04
scalar invariance over time and between groups^c^	4062/3298	0.03	0.92	0.92	0.04

^a^Correlation allowed for item 1 and 5

^b^Autocorrelation allowed

^c^Release of the intercept of item 8 in girls and item 6 in boys over time

To answer the question of how student and teacher culture and composition was associated with gender role attitudes and their development among boys and girls in early adolescence, multigroup correlational analyses with the option *type = complex* in Mplus were conducted. The analysis option provides the opportunity to control for the clustered structure when estimating standard errors with maximum likelihood-estimation procedures with robust standard-error estimates (MLR). Recent studies highlighted the importance of latent modeling and measurement invariance testing when investigating gender role attitudes over time, gender (Ullrich et al., 2022), and countries (e.g., Lomazzi, 2018). Therefore, the *type = complex* option with a latent modeling approach was used rather than multilevel analyses, as the study was concerned with the individual development of boys’ and girls’ traditional gender role attitudes while accounting for different contextual circumstances (Marsh et al., 2009). The analyses were conducted as multigroup models, to examine the differential effects the predictors might have for boys and girls.

When analyzing development, growth curve models are less susceptible to changes between the measurement points, while latent change models allow to examine changes between the measurement points in more detail. In latent change models, differences between the measurement points are estimated as an additional latent component, which allows to investigate interindividual differences in intraindividual changes on a measurement-error-free level (Geiser, 2011). With three measurement points, two latent difference scores were estimated — one captures development over the course of Grade 7 (Diff 2-1), and one captures development between the beginning of Grade 7 and the end of Grade 8 (Diff 3-1). The predictors of interest were then regressed on baseline attitudes at Grade 7 and on the two difference scores to examine how students’ and teachers’ culture and composition were associated with boys’ and girls’ initial gender role attitudes and their development during the first year of secondary education and between Grades 7 and 8. To test whether the prediction of the development for gender role attitudes might differ for the genders, additional difference scores (Diff (Δ) b-g) were conducted to test whether the effects are statistically different for boys and girls. In this context, effects are used in the sense of regression coefficients. Unless stated otherwise, the term “effect” is primarily used as a descriptor of regression effects but not to refer to “causal effects”, which do need further assumptions.

As the school tracks represent different environments concerning their proportion of boys and girls, the school tracks, the perceived peer pressure, and the individual SES were included in the models from the beginning. To answer the first research question regarding how students’ compositional factors were correlated with their initial gender role attitudes and their development during Grades 7 and 8, the compositional school indicators students’ gender role culture, sex composition, and SES composition were included in the baseline change model as predictors of the initial gender role attitudes and the two difference scores (Model 1). To answer the second question regarding how teachers’ gender role attitudes and sex composition were associated with the initial gender role attitudes and their development for boys and girls, these two predictors were included in the baseline change model (Model 2). In the last step, all predictors were included simultaneously to investigate the explanatory power, holding all other factors constant (Model 3).

Concerning missing values, the full information maximum likelihood (FIML) procedure integrated within Mplus 8.4 was used which allows to include participants with missing values. This has the advantage of using all available information of the sample and likewise reduces the risk of bias due to selective dropout (Lüdtke et al., 2007).

## Results

### Student Culture and Composition and the Development of Gender Role Attitudes

To get an impression of the general development of gender role attitudes, Table 3 provides the initial gender role attitudes and the two differences scores over the course of Grade 7 and between Grades 7 and 8. Boys expressed significantly more traditional gender role attitudes than girls (boys: B = 1.766, *SE* = 0.042; girls: B = 1.312, *SE* = 0.026; Diff (Δ) b-g = 0.455, *p* < 0.001). Concerning the development, boys developed significantly more traditional gender role attitudes over the course of Grade 7 (Diff 2-1, B = 0.129, *SE* = 0.022, *p* < 0.001) and between Grades 7 and 8 (Diff 3-1, B = 0.257, *SE* = 0.026, *p* < 0.001). Girls, on the other hand, developed significantly less traditional gender role attitudes over the course of Grade 7 (Diff 2-1, B = −0.085, *SE* = 0.018, *p* < 0.001) and between Grades 7 and 8 (Diff 3-1, B = −0.140, *SE* = 0.023, *p* < 0.001).

**Table 3:** Baseline change model of the general development of male and female adolescents and their differences in the developmental trajectories

	Boys B (S.E.)	Girls B (S.E.)	Diff (Δ) b-g
Gender role attitudes (t1)	1.766 (0.042)	1.312 (0.026)	0.455***
Diff 2-1	0.129 (0.022)***	−0.085 (0.018)***	0.214***
Diff 3-1	0.257 (0.026)***	−0.140 (0.023)***	0.396***

***p < 0.001

To answer the first research question regarding how students’ gender role culture, sex composition, and SES composition were associated with the initial gender role attitudes in Grade 7 and their development over a two-year course, correlational analyses provide initial insights (Table 4). Focusing on boys first, it appears that students’ gender role culture was positively correlated with boys’ gender role attitudes at all three measurement points. This suggests that boys in schools with a more traditional student gender role culture express more individual traditional attitudes. A higher proportion of female students was associated with less traditional attitudes in boys — and, as expected, a more privileged SES composition of students was associated with less traditional gender role attitudes at all three measurement points.

Concerning the students’ gender role culture, a similar pattern emerged for girls. As expected, a more traditional gender role culture among students was associated with more traditional attitudes among girls at the individual level. Despite the assumptions, the students’ sex composition was seemingly unrelated to girls’ gender-role attitudes at all three measurement points. However, in line with the hypothesis, a more privileged SES composition among students was associated with less traditional gender role attitudes among girls at all three measurement points.

In a multivariate perspective, a different pattern emerged regarding the students’ compositional factors and the degree of traditional gender role attitudes at the first measurement point (Table 5). As already shown in the bivariate analyses, the students’ gender role culture was positively associated with boys’ and girls’ initial gender role attitudes (M1, boys: β = 0.461, *SE* = 0.038, *p* < 0.001; girls: β = 0.258, *SE* = 0.054, *p* < 0.001). The difference between these effects were significant, indicating that boys are more sensitive to the students’ gender role culture (M1, Diff (Δ) b-g, B = −0.615, *p* < 0.001). However, the sex composition of the student body was positively associated with boys’ traditional gender role attitudes (M1, β = 0.261, *SE* = 0.031, *p* < 0.001) — a higher proportion of female students was associated with initially more traditional gender role attitudes. For girls, there were no significant effects concerning the students’ sex composition and traditional initial gender role attitudes in a multivariate perspective. The difference between these effects were significant (M1, Diff (Δ) b-g, B = −0.722, *p* < 0.001). Students’ SES composition was only significantly associated with traditional initial gender role attitudes for boys (M1, β = 0.105, *SE* = 0.036, *p* = 0.003), which suggests that a more privileged SES composition is correlated with more traditional gender role attitudes among boys but not for girls. However, the difference between these effects was not significant.

Turning to the implications of students’ compositional factors for the development of gender role attitudes among boys and girls, the results indicate that boys developed in a less traditional direction in contexts with a more traditional student culture over the course of Grade 7 (M1, Diff 2-1, β = −0.189, *SE* = 0.062, *p* = 0.002) and between Grades 7 and 8 (M1, Diff 3-1, β = −0.264, *SE* = 0.059, *p* < 0.001). No significant effects emerged for the students’ sex composition and the development of gender role attitudes in boys. In line with the assumptions, a more privileged student SES composition was negatively associated with the development of traditional gender role attitudes in boys. This indicates that boys developed less traditional gender role attitudes over the course of Grade 7 (M1, Diff 2-1, β = −0.192, *SE* = 0.066, *p* = 0.004) and between Grades 7 and 8 (M1, Diff 3-1, β = −0.158, *SE* = 0.055, *p* = 0.004). Contrary to the assumptions, students’ cultural and compositional factors were unrelated to the development of girls’ gender role attitudes. Comparing the genders, the results indicate that boys are more sensitive to students’ gender role culture over the course of Grade 7 (M1, Diff (Δ) b-g, B = 0.420, *p* = 0.002) and between Grades 7 and 8 (M1, Diff (Δ) b-g, B = 0.486, *p* = 0.027). Compared to girls (M1, Diff (Δ) b-g, B = 0.085, *p* = 0.012), boys also develop less traditional gender role attitudes in schools with a higher SES composition over the course of Grade 7. The effect and difference patterns concerning the students’ compositional indicators and the degree of boys and girls traditional gender role attitudes at the beginning of Grade 7 and the development over the two-year period remained stable when controlling for teacher effects (Model 3).

### Teacher Culture and Composition and the Development of Gender Role Attitudes

To answer how teachers’ gender role culture and sex composition were associated with the initial gender role attitudes of boys and girls, correlation analyses were conducted (Table 4). The initial gender role attitudes among boys and girls in Grade 7 were not significantly correlated with teachers’ gender role culture. However, teachers’ gender role culture was associated with boys’ gender role attitudes at the end of Grade 8, indicating that boys expressed more traditional gender role attitudes in schools with a more traditional teacher culture. The sex composition of the teaching staff was only significantly correlated with boys’ initial gender role attitudes at the beginning of Grade 7 and by the end of Grade 8, indicating that more female teachers in a school were associated with less traditional attitudes in boys. For girls, no significant associations between gender role attitudes and teachers’ gender role culture and sex composition were apparent.

**Table 4:** Correlational analyses separately for boys and girls

		1	2	3	4	5	6	7	8	9	10
1	Gender role attitudes (t1)		0.714***	0.637***	0.306***	−0.053	−0.257***	0.038	0.005	−0.224***	0.441***
2	Gender role attitudes (t2)	0.718***		0.713***	0.319***	−0.069	−0.280***	0.053	0.005	−0.255***	0.354***
3	Gender role attitudes (t3)	0.612***	0.745***		0.275***	−0.061	−0.247***	0.046	−0.019	−0.243***	0.289***
4	Students’ gender role culture	0.358***	0.258***	0.248***		−0.394***	−0.761***	0.158	−0.159	−0.371***	0.084***
5	Students’ sex composition	−0.175***	−0.087*	−0.105**	−0.720***		0.090	−0.088	0.508	0.046	0.019
6	Students’ SES composition	−0.296***	−0.259***	−0.246***	−0.771***	0.412***		−0.108	0.249	0.491***	−0.054*
7	Teachers’ gender role culture	0.100	0.069	0.117**	0.308*	−0.295	−0.155		0.028	−0.029	0.083**
8	Teachers’ sex composition	−0.115*	−0.050	−0.093*	−0.457***	0.633***	0.443***	−0.319*		0.123	0.042
9	Parental SES	−0.262***	−0.242***	−0.239***	−0.365***	0.202***	0.461***	−0.081	0.207***		−0.060**
10	Perceived peer pressure	0.450***	0.354***	0.333***	0.144***	−0.143***	−0.095***	0.104**	−0.127***	−0.095***	

Results for boys below the diagonal and for girls above the diagonal

*p < 0.05. **p < 0.01. ***p < 0.001

The baseline change models confirm the correlational results indicating that teachers’ gender role culture was not associated with initial gender role attitudes among boys and girls in Grade 7 (Table 5, Model 2). The teachers’ sex composition correlated with traditional initial attitudes in boys (M2, β = 0.078, *SE* = 0.038, *p* = 0.038), suggesting that more female teachers in a school are associated with initially more traditional gender role attitudes among boys but not for girls. However, this difference was not significant. When controlling for the students’ cultural and compositional factors (M3), the significant effect of teachers’ sex composition for boys vanishes. For girls, a small significant effect of teachers’ gender role culture on the traditional initial attitudes for girls emerged, indicating that a more traditional teacher culture was associated with initially less traditional attitudes in girls (M3, β = −0.057, *SE* = 0.028, *p* = 0.044).

**Table 5:** Baseline change models to predict the development of gender role attitudes between Grade 7 and 8

	Model 1			Model 2			Model 3		
	Boys β (S.E.)	Girls β (S.E.)	Diff(Δ) b-g	Boys β (S.E.)	Girls β (S.E.)	Diff(Δ) b-g	Boys β (S.E.)	Girls β (S.E.)	Diff(Δ) b-g
Intercept									
Students’ gender role culture	0.461 (0.038)***	0.258 (0.054)***	***				0.461 (0.037)***	0.245 (0.048)***	***
Students’ sex composition	0.261 (0.031)***	0.019 (0.024)	***				0.247 (0.038)***	−0.009 (0.039)	***
Students’ SES composition	0.105 (0.036)**	0.057 (0.039)	n.s.				0.100 (0.032)**	0.037 (0.041)	n.s.
Teachers’ gender role culture				0.015 (0.042)	−0.043 (0.039)	n.s.	−0.021 (0.018)	−0.057 (0.028)*	n.s.
Teachers’ sex composition				0.078 (0.038)*	0.003 (0.041)	n.s.	0.017 (0.021)	0.034 (0.040)	n.s.
Parental SES	−0.104 (0.033)**	−0.093 (0.025)***		−0.136 (0.033)***	−0.124 (0.026)***		−0.104 (0.033)**	−0.090 (0.025)***	
Perceived peer pressure	0.442 (0.022)***	0.444 (0.020)***		0.456 (0.024)***	0.456 (0.020)***		0.444 (0.023)***	0.446 (0.020)***	
Vocational track	0.191 (0.032)***	0.144 (0.035)***		0.288 (0.044)***	0.196 (0.035)***		0.192 (0.033)***	0.149 (0.035)***	
Art track	−0.020 (0.015)	−0.003 (0.029)		−0.016 (0.017)	−0.008 (0.023)		−0.023 (0.014)	−0.008 (0.028)	
Technical track	0.024 (0.025)	0.043 (0.035)		0.110 (0.030)***	0.092 (0.033)**		0.028 (0.027)	0.055 (0.035)	
Diff 2-1									
Students’ gender role culture	−0.189 (0.062)**	0.020 (0.067)	**				−0.247 (0.071)**	−0.021 (0.067)	**
Students’ sex composition	−0.022 (0.057)	−0.002 (0.048)	n.s.				−0.096 (0.079)	−0.039 (0.067)	n.s.
Students’ SES composition	−0.192 (0.066)**	−0.061 (0.083)	*				−0.250 (0.073)**	−0.110 (0.082)	*
Teachers’ gender role culture				0.017 (0.035)	0.051 (0.045)	n.s.	0.044 (0.037)	0.043 (0.049)	n.s.
Teachers’ sex composition				0.019 (0.040)	0.018 (0.048)	n.s.	0.094 (0.055)	0.064 (0.058)	n.s.
Parental SES	−0.049 (0.032)	−0.079 (0.037)*		−0.092 (0.037)*	−0.107 (0.032)**		−0.050 (0.032)	−0.084 (0.037)*	
Perceived peer pressure	−0.096 (0.037)*	−0.116 (0.036)**		−0.100 (0.038)**	−0.116 (0.035)**		−0.094 (0.038)*	−0.117 (0.036)**	
Vocational track	−0.153 (0.047)**	−0.064 (0.065)		−0.176 (0.055)**	−0.054 (0.062)		−0.159 (0.048)**	−0.074 (0.065)	
Art track	−0.041 (0.020)*	−0.001 (0.051)		−0.027 (0.014)	0.006 (0.051)		−0.040 (0.018)*	0.001 (0.048)	
Technical track	−0.073 (0.046)	−0.047 (0.054)		−0.106 (0.046)*	−0.057 (0.049)		−0.093 (0.049)	−0.076 (0.055)	
Diff 3-1									
Students’ gender role culture	−0.264 (0.059)***	−0.048 (0.089)	*				−0.313 (0.055)***	−0.057 (0.097)	*
Students’ sex composition	−0.074 (0.054)	−0.007 (0.049)	n.s.				−0.081 (0.063)	−0.005 (0.062)	n.s.
Students’ SES composition	−0.158 (0.055)**	−0.076 (0.084)	n.s.				−0.181 (0.059)**	−0.081 (0.093)	n.s.
Teachers’ gender role culture				0.075 (0.047)	0.042 (0.058)	n.s.	0.112 (0.042)**	0.045 (0.056)	n.s.
Teachers’ sex composition				−0.026 (0.034)	−0.012 (0.047)	n.s.	0.005 (0.044)	0.004 (0.061)	n.s.
Parental SES	−0.035 (0.041)	−0.088 (0.035)*		−0.043 (0.038)	−0.096 (0.037)*		−0.034 (0.041)	−0.093 (0.036)**	
Perceived peer pressure	−0.152 (0.043)***	−0.192 (0.039)***		−0.168 (0.044)***	−0.194 (0.037)***		−0.156 (0.044)***	−0.195 (0.038)***	
Vocational track	−0.068 (0.040)	−0.113 (0.056)*		−0.135 (0.041)**	−0.107 (0.059)		−0.075 (0.040)	−0.118 (0.058)*	
Art track	−0.037 (0.013)**	−0.068 (0.024)**		−0.033 (0.016)*	−0.059 (0.027)*		−0.032 (0.017)	−0.062 (0.026)*	
Technical track	−0.010 (0.046)	−0.053 (0.038)		−0.076 (0.035)*	−0.046 (0.045)		−0.029 (0.048)	−0.067 (0.043)	

Diff 2-1 and Diff 3-1 = Baseline change difference scores

Diff(Δ) b-g = mean differences in the change values between boys and girls

*p < 0.05. **p < 0.01. ***p < 0.001

When focusing on the implications of teacher effects for the development of gender role attitudes, no significant effects for either boys or girls were found (M2). However, when controlling for the students’ compositional factors, a significant effect of the teachers’ gender role culture on the development of gender role attitudes between Grade 7 and 8 for boys emerged (M3, Diff 3-1, β = 0.112, *SE* = 0.042, *p* = 0.009), indicating that boys are developing in a more traditional direction in schools with a more traditional teacher culture. For girls, no significant effects of the teachers’ cultural and compositional factors on the development of gender role attitudes were apparent. Although some of the effects of teacher composition and culture showed up for only one gender, the effect differences were not statistically significant when controlling for the student culture and composition, suggesting that the factors are not inherently different in their effects between the genders.

### Sensitivity Analyses

Due to the special tracking system in Belgium, the schools provide different, potentially gendered, environments (Van Houtte & Vantieghem, 2020). There are more boys in the technical tracks, and more girls in the art tracks. Thus, gendered contexts arise as a result of the choice behavior, which in turn can reinforce traditional gender roles (cf. Eagly et al., 2020). Therefore, it was important to control for the different tracks in the latent change models from the beginning. However, when performing an analysis without the control variables, stable patterns of the developmental effects were found compared to the presented results of the analysis with the control variables. Differences were evident in boys’ initial attitudes, since SES composition did not significantly correlate with boys’ initial gender role attitudes when not controlling for parental SES and school track. For girls, teachers’ gender role culture was not significantly associated with their initial gender role attitudes. This highlights the importance of controlling for the different school tracks, which can be gendered due to the tracking system.

Due to the longitudinal design, selective dropout cannot always be ruled out. Usually, dropout is rather systematic, with higher achieving and socially positively selected students showing a higher compliance (e.g., Damian et al., 2015). Therefore, it was tested whether the dropout was systematically related to the relevant constructs used in the present study. Examining non-participation in the last wave, the results reveal (Table 7 in the appendix) that panel mortality was statistically stronger among students from less socioeconomically privileged families, students with a migration background and students with more traditional gender role attitudes. As this attrition has a systematic component related to gender role attitudes, it is essential to include all individuals in the analyses and not using missing data strategies such as pairwise or listwise deletion, as these rely on more restrictive assumptions (i.e., missing completely at random, which is not the case here, Graham, 2009). FIML was used to retain all students, which minimizes the risk of bias due to selective dropout and maintains maximal test power, as all available information is used. However, to check for robustness, an analysis was conducted with students who participated at all three measurement points (i.e., relying on casewise deletion strategy; *N* = 4322). The overall pattern of students’ and teachers’ culture and composition and the development of gender role attitudes remained the same for male and female adolescents. In the full model, no significant effects were found regarding teachers’ gender role culture and girls’ initial gender role attitudes. For boys, the negative effect of students’ sex composition and the positive effect of teachers’ gender role culture on the gender role attitude development during Grade 7 turned significant when only considering students who participated at all measurement points. However, it is unclear whether this is due to the more restrictively selected sample, the reduced test power, or even biased due to the assumptions this sample selection makes (missing completely at random which does not apply here, see Table 7).

As previous research presented overall developmental models in the form of growth curve models, the present study used developmental models that were more sensitive to changes between the measurement points. Therefore, baseline change models were used, which offered the opportunity to examine interindividual differences in intraindividual changes on an error-free measurement level (Geiser, 2011). However, to check for robustness, latent growth curve models were examined, which reproduced and supported the results from the baseline change models.

## Discussion

Research has already demonstrated that gender role attitudes develop during adolescence. However, relevant predictors of this development remain a matter of debate. Family background seems to be less relevant for gender role attitude development (Halimi et al., 2021; Ullrich et al., 2022), which is in line with the general knowledge about adolescence when teenagers are distancing themselves from their family context and the school context is becoming more important. Teachers are often overlooked as relevant agents of gender socialization (Retelsdorf et al., 2015; Wolter et al., 2015). Therefore, the present study addressed the question of whether and to what extent students’ gender role culture, sex composition, students’ SES composition, and teachers’ gender role culture and sex composition can predict the development of gender role attitudes among boys and girls between Grades 7 and 8.

Concerning the first research question, the results revealed for boys that students’ traditional gender role culture was positively associated with initially traditional gender role attitudes, but that development was negatively associated with the students’ traditional gender role culture. This indicates that boys in more traditional student environments develop in a less traditional direction. Or, to formulate it differently, boys in traditional environments already expressed more traditional gender role attitudes at the beginning of Grade 7, which then do not develop as strongly in a traditional direction during the first two years of secondary education. Presumably, boys who support traditional gender role attitudes might choose school tracks offering traditionally masculine subjects like technical education and find themselves in a context that does not prompt them to change their gender role attitudes. Conversely, boys who initially expressed less traditional gender role attitudes may developed more traditional attitudes over time in schools with a less traditional student gender role culture. This would suggest that boys’ developmental trajectories are converging. For girls, the students’ traditional gender role culture was positively associated with initially traditional attitudes; however, the development of girls’ gender role attitudes was not associated with the students’ gender role culture.

In a multivariate perspective, boys expressed more traditional initial attitudes in schools with a higher proportion of female students, which could indicate that boys feel the need to stress their masculinity when entering schools with a majority of girls (Van Houtte, 2021). However — contrary to assumptions — the gender distribution was seemingly unrelated to girls’ initial gender role attitudes. The development of gender role attitudes was not associated with the students’ sex composition.

A more privileged student SES composition was associated with initially more traditional gender role attitudes among boys, but it affected gender role attitude development negatively, suggesting that boys in contexts with a more privileged SES composition developed less traditional gender role attitudes. Conversely, boys in schools with a less privileged SES composition expressed initially less traditional gender role attitudes but developed more traditional gender role attitudes during the first two years of secondary education. This suggest a convergence of boys’ developmental trajectories. In Flanders, schools with lower SES compositions are usually offering technical and vocational education, which are gendered subjects with a majority of male students — in such contexts, boys might initially feel the need to support traditional gender role attitudes. Research has shown that following traditional gender role attitudes can be detrimental for students’ academic development (e.g., Hadjar et al., 2012).

For girls, the baseline change models revealed that parental SES was a significant predictor for the development of girls’ traditional gender role attitudes and not students’ SES composition — this contrasts with previous research (Halimi et al., 2021; Ullrich et al., 2022). Girls from more privileged families in SES terms have less traditional initial gender role attitudes and their traditional attitudes decline even more so in young adolescence.

Concerning the effects of teachers’ culture and composition, boys show slightly more traditional initial gender role attitudes in schools with more female teachers, when not controlling for students’ compositional and cultural indicators. This suggests that boys might feel threatened by a higher proportion of female teachers, which may prompt them to stress their masculinity more and foster traditional gender role attitudes. Girls show less traditional gender role attitudes in schools with a more traditional teacher gender role culture. In a developmental perspective, boys develop more traditional gender role attitudes in schools with a more traditional gender role culture among teachers. Teachers can consciously or unconsciously transmit their gendered expectations. Teachers seem to be a relevant normative reference — and due to gendered expectations or role modeling, teachers can enhance and reproduce society’s gender distribution (Retelsdorf et al., 2015; Wolter et al., 2015).

Comparing boys and girls, the results reveal that boys are more susceptible to the student culture and composition with regard to the development of gender role attitudes. This is in line with research that has shown that boys are more sensitive to gender conformity pressures (e.g., Vantieghem & Van Houtte, 2015). However, the effects of teachers’ culture and composition differed not statistically significant between boys’ and girls’ gender role attitude development, suggesting gender similarities between the genders (Hyde, 2014). Although girls seem to be less susceptible to the students’ environment, less traditional or more egalitarian gender roles among students and teachers should be especially relevant for them with a view to ensuring they are equally supported in pursuing their interests and participating in school subjects (Wolter et al., 2015).

### Limitations

Previous research concerning the measurement of gender role attitudes indicated difficulties in reaching measurement invariance across time points, genders (Ullrich et al., 2022), and countries (Lomazzi & Seddig, 2020). Gender role attitudes are multidimensional with topics such as gendered responsibilities of working and private life, traditional masculinity, and gender perceptions of boys and girls. All these facets of gender role attitudes were included in the scales and EFA and CFA testing confirmed the unidimensionality of the scale (for a detailed examination of the scale, see Halimi et al., 2018). However, due to the multidimensionality and the development over time, challenges can arise when testing the scale for measurement invariance (e.g., Ullrich et al., 2022). This difficulty was also present here, since partial measurement invariance had to be applied to yield scalar invariance over time and between boys and girls. This highlights the importance of measuring gender role attitudes with latent scales to ensure comparability over time and between genders. To check the robustness of the gender role attitudes scale, the latent modeling and measurement invariance over time and genders were cross-checked with students who participated at all three measurement points (*N* = 4322). Partial scalar invariance was reached over time and between genders with the same adjustments that had to be applied to the complete sample (RMSEA = 0.04, CFI = 0.92, TLI = 0.92, SRMR = 0.05).

By examining developmental trajectories, statistical artifacts such as regression to the mean cannot always be ruled out. However, modeling traditional gender role attitudes with a latent factor structure should have improved the likelihood of detecting differential developments and limit the risk of regression to the mean artifacts, since the measurement error variance is lower compared to a manifest model (Becker, 2009).

The results showed a significant effect of parental SES on the development of girls’ gender role attitudes. So far, in overall growth curve models no significant effect of parental SES on the development of gender role attitudes was found (Halimi et al., 2021; Ullrich et al., 2022). Moreover, previous research has shown that socioeconomic background is associated with boys’ and girls’ cognitive abilities and that this effect is confounded when examining the development of gender role attitudes (Ullrich et al., 2022). Yet, the present dataset did not permit us to consider students’ cognitive abilities.

The present study addressed the individual development of gender role attitudes. However, societal gender roles develop as well (Scarborough et al., 2019). It was not possible to consider cohort effects related to individual gender role attitude development that might go beyond the influence of the school context. As such, this is an interesting aspect for future research and the present study can serve as a central point of reference in this endeavor.

### Implications

This study has key implications for future research. Methodologically, this study stresses the importance of measurement invariance testing for gender role attitudes scales over time and between genders. Even though this study focused on a short period of time, partial measurement invariance had to be applied to reach scalar invariance over time and between genders.

For future research, the study highlights the importance of contextual school factors for the development of gender role attitudes. Teachers can act as important socialization agents if they concentrate on the process of (implicitly or explicitly) reproducing gender divisions. As gender role attitudes change during adolescence, teachers can actively promote non-stereotypical interests and model non-stereotypical behaviors. Promoting more open attitudes is associated with higher general interest profiles (Ehrtmann et al., 2019), performance development (Ehrtmann & Wolter, 2018), and general school achievement (Hadjar et al., 2012) for girls and boys. Therefore, it would be interesting to shed light on teachers’ implicit and explicit gendered expectations and the mechanisms in reproducing gendered divisions in school.

Another interesting aspect for future research may be the variance in gender role attitudes within schools. Some schools may have very homogeneous attitudes towards gender roles, but in other schools, students and teachers may have very different gender role attitudes. This would presumably have different effects on students’ gender role attitudes (cf. Van Houtte, 2023).

## Conclusion

Gender role attitudes develop during adolescence; however, there is very little research that examines the relevance of the school’s context on the development of gender role attitudes and teachers — as important socialization agents — are rarely taken into account. The present study examined the development of gender role attitudes of young adolescent boys’ and girls’ after transitioning to secondary education. For the first time this study examined how students’ and teachers’ cultural and compositional characteristics can explain the development of gender role attitudes during emerging adolescence. During adolescence gender related constructs are especially salient and students and teachers are regarded as important socialization agents. The results highlighted that the initial gender role attitudes and the development vary with cultural and compositional characteristics of the students and teachers with an emphasis on students’ contextual characteristics. Thereby, especially male students seem susceptible to contextual characteristics, which is in line with previous research showing that especially boys are sensitive to peer pressure. Studying the development of gender role attitudes is important for understanding gender inequalities as gender-related constructs are particularly salient during adolescence, when adolescents are discovering their own gender identity. In future research, it would be interesting to examine the development of gender role attitudes throughout the course of adolescence as the compositional characteristics of teachers and students may become more important during adolescence. The results of this study highlight the importance of examining students’ and teachers’ gender culture and composition.

## Data Availability

Materials and analysis code for this study are available by emailing the corresponding author. The dataset is available by emailing the second author. This study was not preregistered.

## Appendix

Tables 6, 7

**Table 6:** Baseline change models to predict the development of gender role attitudes between Grade 7 and 8

	Model 1			Model 2			Model 3		
	Boys B (S.E.)	Girls B (S.E.)	Diff(Δ) b-g	Boys B (S.E.)	Girls B (S.E.)	Diff(Δ) b-g	Boys B (S.E.)	Girls B (S.E.)	Diff(Δ) b-g
Intercept									
Students’ gender role culture	1.305 (0.076)***	0.690 (0.118)***	−0.615***				1.304 (0.080)***	0.655 (0.118)***	−0.649***
Students’ sex composition	0.811 (0.076)***	0.089 (0.114)	−0.722***				0.769 (0.096)***	−0.041 (0.185)	−0.810***
Students’ SES composition	0.084 (0.027)**	0.033 (0.023)	−0.051				0.080 (0.024)**	0.022 (0.024)	−0.058
Teachers’ gender role culture				0.214 (0.579)	−0.483 (0.447)	−0.697	−0.296 (0.249)	−0.636 (0.306)*	−0.340
Teachers’ sex composition				0.360 (0.166)*	0.015 (0.200)	−0.345	0.080 (0.092)	0.165 (0.200)	0.085
Parental SES	−0.038 (0.013)**	−0.025 (0.007)***		−0.049 (0.013)***	−0.034 (0.008)***		−0.037 (0.013)**	−0.025 (0.007)**	
Perceived peer pressure	1.024 (0.077)***	0.920 (0.060)***		1.050 (0.080)***	0.944 (0.063)***		1.028 (0.079)***	0.925 (0.061)***	
Vocational track	0.338 (0.046)***	0.244 (0.056)***		0.506 (0.066)***	0.333 (0.057)***		0.340 (0.048)***	0.252 (0.055)***	
Art track	−0.176 (0.116)	−0.012 (0.133)		−0.139 (0.141)	−0.035 (0.107)		−0.204 (0.101)*	−0.037 (0.129)	
Technical track	0.036 (0.039)	0.061 (0.049)		0.167 (0.045)***	0.129 (0.047)**		0.043 (0.041)	0.077 (0.049)	
Diff 2-1									
Students’ gender role culture	−0.382 (0.125)**	0.038 (0.125)	0.420**				−0.501 (0.149)**	−0.040 (0.127)	0.461**
Students’ sex composition	−0.049 (0.127)	−0.008 (0.159)	0.041				−0.214 (0.181)	−0.128 (0.215)	0.086
Students’ SES composition	−0.109 (0.035)**	−0.025 (0.033)	0.085*				−0.142 (0.037)***	−0.045 (0.032)	0.098*
Teachers’ gender role culture				0.171 (0.355)	0.403 (0.343)	0.232	0.444 (0.366)	0.334 (0.372)	−0.110
Teachers’ sex composition				0.062 (0.134)	0.062 (0.165)	0.001	0.312 (0.193)	0.222 (0.191)	−0.091
Parental SES	−0.013 (0.008)	−0.015 (0.007)*		−0.024 (0.010)*	−0.021 (0.006)**		−0.013 (0.008)	−0.016 (0.007)*	
Perceived peer pressure	−0.159 (0.061)*	−0.168 (0.054)**		−0.166 (0.062)**	−0.170 (0.053)**		−0.156 (0.063)**	−0.170 (0.055)**	
Vocational track	−0.193 (0.059)**	−0.076 (0.077)		−0.222 (0.067)**	−0.065 (0.074)		−0.202 (0.061)**	−0.088 (0.076)	
Art track	−0.257 (0.128)*	−0.002 (0.160)		−0.172 (0.086)*	0.020 (0.165)		−0.256 (0.107)*	0.003 (0.150)	
Technical track	−0.080 (0.051)	−0.046 (0.053)		−0.116 (0.050)*	−0.057 (0.048)		−0.102 (0.054)	−0.075 (0.054)	
Diff 3-1									
Students’ gender role culture	−0.586 (0.132)***	−0.100 (0.187)	0.486*				−0.695 (0.129)***	−0.118 (0.205)	0.577*
Students’ sex composition	−0.179 (0.136)	−0.024 (0.177)	0.155				−0.198 (0.155)	−0.016 (0.226)	0.182
Students’ SES composition	−0.098 (0.033)**	−0.034 (0.037)	0.064				−0.114 (0.036)**	−0.036 (0.041)	0.077
Teachers’ gender role culture				0.825 (0.538)	0.372 (0.509)	−0.453	1.233 (0.452)**	0.391 (0.492)	−0.841
Teachers’ sex composition				−0.096 (0.123)	−0.047 (0.182)	0.049	0.020 (0.159)	0.015 (0.232)	−0.005
Parental SES	−0.010 (0.012)	−0.019 (0.008)**		−0.012 (0.011)	−0.021 (0.008)**		−0.010 (0.012)	−0.020 (0.008)*	
Perceived peer pressure	−0.276 (0.080)**	−0.310 (0.069)***		−0.304 (0.082)***	−0.313 (0.066)***		−0.283 (0.082)**	−0.314 (0.067)***	
Vocational track	−0.094 (0.055)	−0.148 (0.074)*		−0.186 (0.055)**	−0.142 (0.079)		−0.104 (0.054)	−0.155 (0.076)*	
Art track	−0.258 (0.060)***	−0.230 (0.056)***		−0.226 (0.079)**	−0.207 (0.068)**		−0.225 (0.087)*	−0.217 (0.062)***	
Technical track	−0.011 (0.055)	−0.057 (0.041)		−0.091 (0.041)*	−0.050 (0.049)		−0.035 (0.058)	−0.073 (0.047)	

Diff 2-1 and Diff 3-1 = Baseline change difference scores

Diff(Δ) b-g = mean differences in the change values between boys and girls

**p* < 0.05. ***p* < 0.01. ****p* < 0.001

**Table 7:** Logistic regression of parental SES, migration background, gender and gender role attitudes on non-participation in the last wave

	B (S.E.)
Parental SES	−0.125 (0.020)***
Migration background	0.348 (0.042)***
Sex	−0.018 (0.079)
Gender role attitudes (t1)	0.139 (0.082)
Gender role attitudes (t2)	0.193 (0.079)**

*N* = 5683

***p* < 0.01. ****p* < 0.001
